# Wnt signaling in cancer: therapeutic targeting of Wnt signaling beyond β-catenin and the destruction complex

**DOI:** 10.1038/s12276-020-0380-6

**Published:** 2020-02-10

**Authors:** Youn-Sang Jung, Jae-Il Park

**Affiliations:** 1 Department of Experimental Radiation Oncology, Division of Radiation Oncology, The University of Texas MD Anderson Cancer Center, Houston, TX 77030 USA; 2 Graduate School of Biomedical Sciences, The University of Texas MD Anderson Cancer Center, Houston, TX 77030 USA; 3 Program in Genetics and Epigenetics, The University of Texas MD Anderson Cancer Center, Houston, TX 77030 USA

**Keywords:** Cancer, Drug discovery

## Abstract

Wnt/β-catenin signaling is implicated in many physiological processes, including development, tissue homeostasis, and tissue regeneration. In human cancers, Wnt/β-catenin signaling is highly activated, which has led to the development of various Wnt signaling inhibitors for cancer therapies. Nonetheless, the blockade of Wnt signaling causes side effects such as impairment of tissue homeostasis and regeneration. Recently, several studies have identified cancer-specific Wnt signaling regulators. In this review, we discuss the Wnt inhibitors currently being used in clinical trials and suggest how additional cancer-specific regulators could be utilized to treat Wnt signaling-associated cancer.

## Cancer: A search for safer signaling targets

More effective treatments for cancer could be developed by targeting signaling pathway regulators that are expressed solely in cancer cells. Disruption to a major signaling pathway known as Wnt, which is involved in processes including cell proliferation, tissue homeostasis and tissue regeneration, is now recognized as a significant contributor to the development of certain cancers. Jae-Il Park and Youn-Sang Jung at the University of Texas MD Anderson Cancer Center, Houston, USA, reviewed recent research into Wnt signaling in cancer and possible therapies. Scientists have developed Wnt inhibitors for cancer treatment, but these have detrimental side effects including skeletal degeneration and abdominal pain. New studies suggest there are Wnt signaling regulators that are specifically expressed in cancer cells, which may prove to be more effective drug targets than blocking Wnt signaling as a whole.

## Introduction

Wnt signaling orchestrates various biological processes, such as cell proliferation, differentiation, organogenesis, tissue regeneration, and tumorigenesis^1–5^. Classically, Wnt signaling is divided into β-catenin-dependent (canonical, Wnt/β-catenin pathway) and β-catenin-independent (noncanonical, Wnt/planar cell polarity [PCP] and calcium pathway) signaling^6,7^. Canonical Wnt signaling mainly regulates cell proliferation, and noncanonical Wnt signaling controls cell polarity and movement. However, this terminological distinction is unclear, and has been questions by studies proposing the involvement of both β-catenin-dependent and β-catenin-independent Wnt signaling in tumorigenesis^8^. For instance, APC and β-catenin are not only involved in cell proliferation but have also been linked to cell-to-cell adhesion^9^. In this review, we will discuss an ongoing effort to inhibit Wnt signaling and suggest potential approaches to target Wnt signaling for cancer therapies proposed from recent studies.

## Wnt signaling and clinical trials in human cancers

β-Catenin is a crucial signaling transducer in Wnt signaling^10,11^. The β-catenin protein destruction complex composed of adenomatous polyposis coli (APC), casein kinase 1 (CK1), glycogen synthase kinase 3α/β (GSK-3α/β), and AXIN1 tightly controls β-catenin via phosphorylation-mediated proteolysis^10,12–16^. In this section, we briefly describe how genetic alterations of Wnt signaling contribute to tumorigenesis and introduce recent clinical trials that have aimed to inhibit Wnt signaling for cancer treatment.

### The β-catenin destruction complex

Colorectal cancer (CRC) is the representative of human cancer caused by Wnt signaling hyperactivation^17,18^. CRC displays a high mutation frequency in *APC* (~70%)^19–21^. In 1991, *APC* mutation was identified as the cause of hereditary colon cancer syndrome, also called familial adenomatous polyposis^22^. APC forms the β-catenin destruction complex in association with CK1, AXIN1, and GSK-3 and interacts with β-catenin^15,23,24^. This protein destruction complex downregulates β-catenin through phosphorylation and ubiquitin-mediated protein degradation^10,12–16^. Genetic mutations causing the loss of function of the destruction complex or gain of function of β-catenin lead to nuclear translocation of β-catenin, resulting in T-cell factor (TCF)4/β-catenin-mediated transactivation of Wnt target genes^25,26^. The Vogelstein group established a multistep tumorigenesis model of CRC. *APC* mutation is an early event that initiates CRC adenoma^27^. CRC progression also requires additional genetic alterations in *KRAS*, *PI3K*, *TGF-β*, *SMAD4*, and *TP53*^27^. Moreover, epigenetic silencing of negative regulators of Wnt signaling was also frequently found in the absence of *APC* mutations^28,29^. APC is a multifunctional protein. In addition to its role in β-catenin degradation, APC binds to actin and actin-regulating proteins^30–33^, which controls the interaction between E-cadherin and α-/β-catenin and various physiological processes, including migration and chromosomal fidelity^34–38^. Importantly, recent studies revealed that *APC* mutation is insufficient to fully activate Wnt signaling. Furthermore, even if *APC* is mutated, mutant APC still negatively regulates β-catenin to some extent^39,40^, which will be discussed later.

AXIN1 is a multidomain scaffolding protein that forms the β-catenin destruction complex in association with APC, CK1, and GSK3^10,41,42^. In human cancer, *AXIN1* mutations are scattered throughout the whole coding sequence of the *AXIN1* gene^43,44^, which results in disassembly of the β-catenin destruction complex. As a priming kinase, CK1 initially phosphorylates β-catenin (Ser45), which induces the sequential phosphorylation of β-catenin by GSK3. Subsequently, phosphorylated β-catenin is recognized and degraded by E3 ubiquitin ligase (β-TrCP)^10,12–16^. GSK3 is a serine/threonine kinase that phosphorylates three serine/threonine residues of β-catenin (Ser33, Ser37, and Thr41)^45,46^. Since GSK3 does not bind to β-catenin directly, AXIN1 and APC facilitate the interaction of GSK3 with β-catenin^47,48^. Moreover, unphosphorylated AXIN1 shows a low binding affinity to β-catenin, which is increased by phosphorylation of AXIN1 via GSK3 kinase activity^49,50^. Low-density lipoprotein receptor-related protein 5/6 (LRP5/6) coreceptor is also phosphorylated by CK1 and GSK3, leading to the recruitment of AXIN1 to the membrane^51–53^.

### WNT ligands and receptors

Under physiological conditions, Wnt signaling is activated by the binding of secreted WNT ligands to LRP5/6 coreceptors and frizzled (FZD) receptors^54^, which induces the recruitment of the protein destruction complex to the LRP receptors and the subsequent phosphorylation of the Ser/Pro-rich motif of the LRP cytoplasmic domain via GSK3^15,55,56^. This event activates dishevelled (DVL) and inhibits GSK3, resulting in the inhibition of the phosphorylation-mediated β-catenin protein degradation and the stabilization/accumulation of β-catenin. Then, β-catenin undergoes nuclear translocation and transactivates Wnt target genes^57^. The secretion of WNT ligands mainly depends on acylation by Porcupine (PORCN)^58,59^. PORCN is a membrane-bound O-acyltransferase that mediates the palmitoylation of WNT ligands to induce their secretion. In line with this observation, PORCN shows increased genetic alterations in various human cancers, including esophageal, ovarian, uterine, lung, and cervical cancers^60^.

### Mutations in *CTNNB1*/β-catenin

Unlike CRC, in which the *APC* gene is frequently mutated, the *CTNNB1* gene encoding β-catenin is predominantly mutated in hepatocellular carcinoma, endometrial cancer, and pancreatic cancer^61–63^. The *CTNNB1*/β-catenin gene harbors 16 exons. β-Catenin is mainly composed of three domains (N-terminal [~150 aa], armadillo repeat [12 copies; 550 aa], and C-terminal [~100 aa]). The N-terminal domain contains the phosphorylation sites for GSK3 and CK1^12,14,45,46^, which induces β-TrcP-mediated β-catenin degradation. The C-terminal domain is involved in transactivation of Wnt target genes via TCF/LEF interactions^25,64–66^. The armadillo repeat domain interacts with various proteins, including E-cadherin, APC, AXIN1, and PYGOs/Pygopus^67,68^. In human cancer, the phosphorylation sites (Ser/Thr) in the N-terminal domain of *CTNNB1*/β-catenin are mutational hotspots^14,69,70^, demonstrating that escape from destruction complex-mediated β-catenin protein degradation is a key process for Wnt signaling-induced tumorigenesis.

## Therapeutic targeting of Wnt/β-catenin signaling

**Fig. 1:**
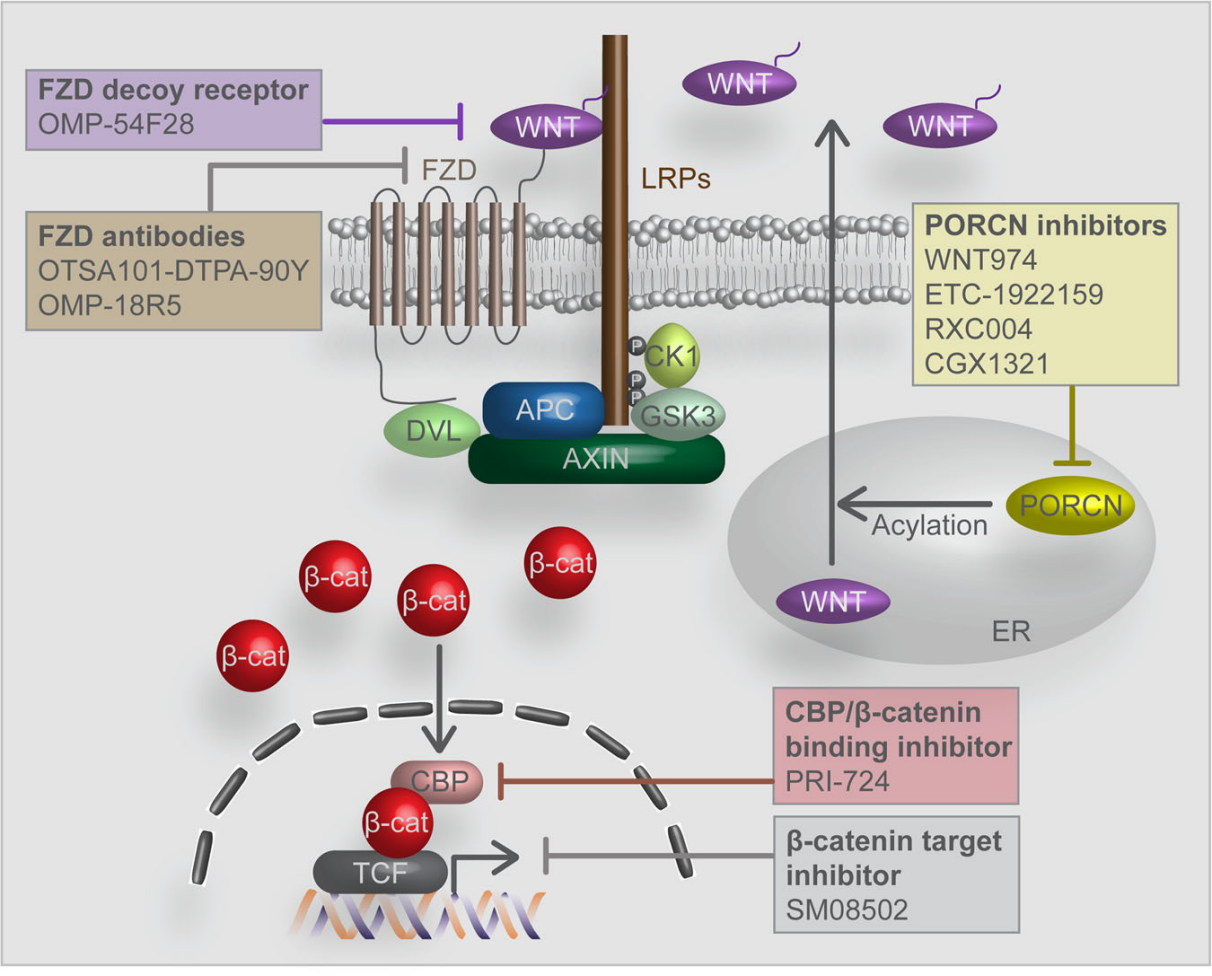
Wnt/β-catenin signaling inhibitors in current and past clinical trials (also see Table **1**).

To suppress WNT ligands or receptors for cancer treatment, PORCN inhibitors, WNT ligand antagonists, and FZD antagonists/monoclonal antibodies have been examined in clinical trials of various Wnt signaling-associated human cancers (Table 1 and Fig. 1).

**Table 1:** Wnt/β-catenin signaling inhibitors in current and past clinical trials.

Drug	Mechanism of action	Cancer type	Phase	Identifier
*WNT974 (with LGX818 and Cetuximab)	PORCN inhibitor	Metastatic CRC	Phase 1	NCT02278133
WNT974	PORCN inhibitor	Squamous cell cancer Head&Neck	Phase 2	NCT02649530
WNT974	PORCN inhibitor	Pancreatic cancer BRAF mutant CRC Melanoma TNBC H&N Squamous cell cancer (cervical, esophageal, lung)	Phase 1	NCT01351103
ETC-1922159	PORCN inhibitor	Solid tumor	Phase 1	NCT02521844
RXC004	PORCN inhibitor	Solid tumor	Phase 1	NCT03447470
CGX1321	PORCN inhibitor	Colorectal adenocarcinoma Gastric adenocarcinoma Pancreatic adenocarcinoma Bile duct carcinoma HCC Esophageal carcinoma Gastrointestinal cancer	Phase 1	NCT03507998
*CGX1321 (with Pembrolizumab)	PORCN inhibitor	Solid tumors GI cancer	Phase 1	NCT02675946
OTSA101-DTPA-90Y	FZD10 antagonist	Sarcoma, Synovial	Phase 1	NCT01469975
*OMP-18R5 (with Docetaxel)	Monoclonal antibody against FZD receptors	Solid tumors	Phase 1	NCT01957007
OMP-18R5	Monoclonal antibody against FZD receptors	Metastatic breast cancer	Phase 1	NCT01973309
OMP-18R5	Monoclonal antibody against FZD receptors	Solid tumors	Phase 1	NCT01345201
*OMP-18R5 (with Nab-Paclitaxel and Gemcitabine)	Monoclonal antibody against FZD receptors	Pancreatic cancer Stage IV pancreatic cancer	Phase 1	NCT02005315
*OMP-54F28 (with Sorafenib)	FZD8 decoy receptor	Hepatocellular cancer Liver cancer	Phase 1	NCT02069145
*OMP-54F28 (with Paclitaxel & Carboplatin)	FZD8 decoy receptor	Ovarian cancer	Phase 1	NCT02092363
*OMP-54F28 (with Nab-Paclitaxel and Gemcitabine)	FZD8 decoy receptor	Pancreatic cancer Stage IV pancreatic cancer	Phase 1	NCT02050178
OMP-54F28	FZD8 decoy receptor	Solid tumors	Phase 1	NCT01608867
PRI-724	CBP/β-catenin antagonist	Advanced pancreatic cancer Metastatic pancreatic cancer Pancreatic adenocarcinoma	Phase 1	NCT01764477
PRI-724	CBP/β-catenin antagonist	Advanced solid tumors	Phase 1	NCT01302405
PRI-724	CBP/β-catenin antagonist	Acute myeloid leukemia Chronic myeloid leukemia	Phase 2	NCT01606579
*PRI-724 (with Leucovorin Calcium, Oxaliplatin, or Fluorouracil)	CBP/β-catenin antagonist	Acute myeloid leukemia Chronic myeloid leukemia	Phase 2	NCT02413853
SM08502	β-catenin-controlled gene expression inhibitor	Solid tumors	Phase 1	NCT03355066

(i) PORCN inhibitors

WNT974 (LGK974; NIH clinical trial numbers [clinicaltrials.gov]: NCT02278133, NCT01351103, and NCT02649530), ETC-1922159 (ETC-159; NCT02521844), RXC004 (NCT03447470), and CGX1321 (NCT02675946 and NCT03507998) are orally administered PORCN inhibitors that commonly bind to PORCN in the endoplasmic reticulum^71–74^. Therefore, PORCN inhibitors block the secretion of WNT ligands through inhibition of posttranslational acylation of WNT ligands. However, similar to other cancer therapies targeting the Wnt pathway, skeletal side effects such as impairment of bone mass and strength and increase in bone resorption were caused by PORCN inhibitor administration^75^.

(ii) SFRP and SFRP peptides

SFRPs (secreted frizzled-related proteins) are soluble proteins. Given the structural homology of SFRPs with the WNT ligand-binding domain in the FZD receptors, SFRPs function as antagonists that bind to WNT ligands and prevent Wnt signaling activation^76–78^. Indeed, SFRPs or SFRP-derived peptides showed tumor suppressive activity in preclinical models^79,80^.

(iii) FZD antagonist/monoclonal antibody

Vantictumab (OMP-18R5; NIH clinical trial numbers [clinicaltrials.gov]; NCT02005315, NCT01973309, NCT01345201, and NCT01957007) is a monoclonal antibody directly binding to FZD receptors, which blocks the binding of WNT ligands to FZD 1, 2, 5, 7, and 8^81^. Ipafricept (OMP-54F28; NIH clinical trial numbers: NCT02069145, NCT02050178, NCT02092363, and NCT01608867) is a recombinant fusion protein that binds to a human IgG1 Fc fragment of FZD8^82,83^. These reagents negatively regulate Wnt/β-catenin signaling through their direct binding to FZD, which thereby disrupts the function of LRPs/FZDs. Alternatively, a way of targeting and killing cancer cells that express high FZD receptors is also being examined. OTSA101 is a humanized monoclonal antibody against FZD10. OTSA101-DTPA-90Y (NIH clinical trial number [clinicaltrials.gov] NCT01469975) is labeled with a β-radiation delivering-yttrium Y90 for OSTA101^84^. OTSA101-DTPA-90Y selectively killed cancer cells highly expressing FZD10. The side effects of vanctumab include tiredness, diarrhea, vomiting, constipation, and abdominal pain. Vantictumab and ipafricept might also cause bone metabolism disorders^81,82^.

(iv) Targeting of LRP degradation and FZD endocytosis

Salinomycin, rottlerin, and monensin induce the phosphorylation of LRP6, resulting in the degradation of LRP6^85–87^. In addition, niclosamide promotes FZD1 endocytosis, which downregulates WNT3A-stimulated β-catenin stabilization^88^. However, these reagents do not specifically target cancer-specific molecules, leading to side effects, including itchiness, abdominal pain, vomiting, dizziness, skin rash, and unpleasant taste^88,89^.

Given that the β-catenin protein destruction complex plays a crucial role in negatively regulating Wnt signaling, the restoration of this protein destruction complex may effectively inhibit Wnt/β-signaling. Tankyrase interacts with and degrades AXIN via ubiquitin-mediated proteasomal degradation^90–92^. Tankyrase inhibitors have been developed^90,93–95^. Indeed, Tankyrase inhibitors have been shown to negatively regulate Wnt signaling in *APC*-mutated cancer cells^93–95^.

(i) Tankyrase inhibitors

Tankyrase inhibitors downregulate β-catenin stabilization. In preclinical studies, Tankyrase inhibitors, including XAV939, JW-55, RK-287107, and G007-LK, stabilized AXIN by inhibiting the poly-ADP-ribosylating enzyme Tankyrase^90–92^. However, currently, no clinical trials are being conducted with Tankyrase inhibitors.

(ii) CK1 agonist

Pyrvinium is an FDA-approved anti-helminthic drug. Pyrvinium binds to CK1 family members in vitro and promotes CK1 kinase activity^96^.

β-Catenin contributes to tumorigenesis via transactivation of Wnt target genes such as *CCND1*, *CD44*, *AXIN2*, and *MYC*^97–100^. Thus, approaches inhibiting either β-catenin transcriptional activity or β-catenin target genes have been developed as potential therapeutic candidates for Wnt signaling-associated cancers (Table 1).

(i) Inhibitors of β-catenin transcriptional activity

β-Catenin/CBP binds to WRE (Wnt-responsive element; 5′-CTTTGA/TA/T-3′) and activates target gene transcription^101,102^. PRI-724 (ICG-001; NIH clinical trial numbers: NCT01302405, NCT02413853, NCT01764477, and NCT01606579) inhibits the interaction between CBP and β-catenin and prevents transcription of Wnt target genes^103^. Moreover, various inhibitors of TCF/LEF and β-catenin interactions have been identified and evaluated in preclinical settings^104^.

To transactivate Wnt target genes, β-catenin forms a transcriptional complex with coactivators, including BCL9 and PYGO^105,106^, which is inhibited by carnosic acid, compound 22, and SAH-BLC9^107,108^. In addition, Pyrvinium downregulates Wnt transcriptional activity through the degradation of PYGO^96^.

(ii) Inhibitor of Wnt target genes

SM08502 (NIH clinical trial number NCT03355066) is a small molecule that inhibits serine and arginine-rich splicing factor (SRSF) phosphorylation and disrupts spliceosome activity. Upon oral administration, SM08502 was shown to downregulate Wnt signaling-controlled gene expression.

(iii) Proteasomal degradation of β-catenin

MSAB (methyl 3-[(4-methylphenyl)sulfonyl]amino-benzoate) binds to β-catenin and facilitates the ubiquitination-mediated proteasomal degradation of β-catenin^108,109^.

However, since β-catenin controls various physiological processes, downregulation of the transcriptional activity β-catenin was shown to induce diarrhea, hypophosphatemia, reversible elevated bilirubin, nausea, fatigue, anorexia, and thrombocytopenia^59,110^.

## Additional layers of Wnt/β-catenin signaling activation

### The β-catenin paradox

Wnt signaling hyperactivation by mutations in β-catenin destruction complex components or β-catenin itself contributes to tumorigenesis. In addition to *APC* mutations, β-catenin can be further activated by additional layers of regulation^39,40,111–117^, which demonstrated the complexity of Wnt signaling deregulation in cancer. Accumulating evidence supports the notion that additional regulatory processes contribute to Wnt signaling hyperactivation in cancer, as demonstrated in the following examples. (a) Mutant APC is still able to downregulate β-catenin^39,40^. (b) Even in the presence of APC mutations, blockade of WNT ligands triggers apoptosis or growth inhibition^40,113,118^. (c) β-Catenin fold induction is essential for the activation of β-catenin target genes^119–121^. (d) Increased AXIN1 by Tankyrase inhibitor suppresses cell proliferation of cancer cells where Wnt/β-catenin signaling is genetically hyperactive^43,90,93,95,122^. (e) Mutations in RNF43 and ZNRF3 E3 ligases that degrade Wnt receptors contribute to tumor development^111,115^. (f) Ras/MAPK signaling is also required for Wnt signaling activation^112,123^. These reports suggest that additional layers further enhance Wnt signaling activation in cancer.

### The lysosome and Wnt signaling

The lysosome contains 40 types of hydrolytic enzymes, including cathepsins, which become active under acidic conditions^124^. Lysosomal hydrolytic enzymes mediate the degradation of phagocytosed material and proteolysis of cytosolic proteins through fusion with the multivesicular body (MVB). Luminal acidification of the lysosome is required for lysosomal protein degradation, which is mainly controlled by vacuolar H^+^ transporters in the lysosomal membrane^125^.

Recently, this classical view of lysosomal functions has evolved into new perspectives highlighting the roles of lysosomes in transcriptional regulation and metabolic homeostasis^126^. In human cancer, lysosomal dysfunction is involved in the generation of building blocks, cell proliferation, metastasis, angiogenesis, and tumor suppressor degradation^39,127^.

It has been reported that Wnt signaling is involved in the endocytosis-mediated formation of the LRP signalosome into the MVB^123,128^. GSK3 in the LRP signalosome is sequestered into the MVB, which leads to an increase in the level of cytosolic β-catenin and inhibition of Wnt signaling^129^. However, decreased GSK3 kinase activity by MVB sequestration lasts approximately 1 h^129,130^. Moreover, it is unclear how sequestrated APC, GSK3, AXIN, and CK1 in MVB are processed. A recent study showed that clathrin-mediated endocytosis is required for Wnt signaling activation, which is inhibited by APC^131^. These studies suggest that vesicular acidification and trafficking also play crucial roles in controlling Wnt/β-catenin signaling through modulation of the protein destruction complex. Next, we discuss how APC is deregulated for Wnt signaling hyperactivation in cancer cells.

Wnt signaling activation requires v-ATPase (vacuolar H^+^-ATPase; an electrogenic H^+^ transporter)^125,132,133^. Previous studies imply that in cancer cells, the upregulation of v-ATPase activity might trigger abnormal Wnt/β-catenin signaling and contribute to Wnt signaling-dependent tumorigenesis. Growing evidence has demonstrated the effect of v-ATPase on various oncogenic processes, including cellular signaling, survival, drug resistance, and metastasis^125,134^. Moreover, the v-ATPase subunits are highly expressed in colorectal, breast, prostate, liver, ovarian, and pancreatic cancer cells^135–138^. The v-ATPase complex is composed of the V1 domain (in the cytosol) and V0 domain (on the membrane)^139,140^. The V1 domain shows reversible disassociation from the V0 domain under physiological conditions, including glucose concentration, starvation of amino acids, and infection of cells by influenza virus^141–144^. Recently, TMEM9 (transmembrane protein 9) was identified as an activator of v-ATPase and is highly expressed in cancer^39^. TMEM9 amplifies Wnt signaling through the v-ATPase-mediated lysosomal protein degradation of APC^39^. Given that TMEM9 is highly expressed in CRC cells and that *Tmem9* knockout mice are also viable^39^, molecular targeting of TMEM9 may selectively suppress Wnt signaling activity in cancer cells.

## Novel therapeutic target: v-ATPase

**Fig. 2:**
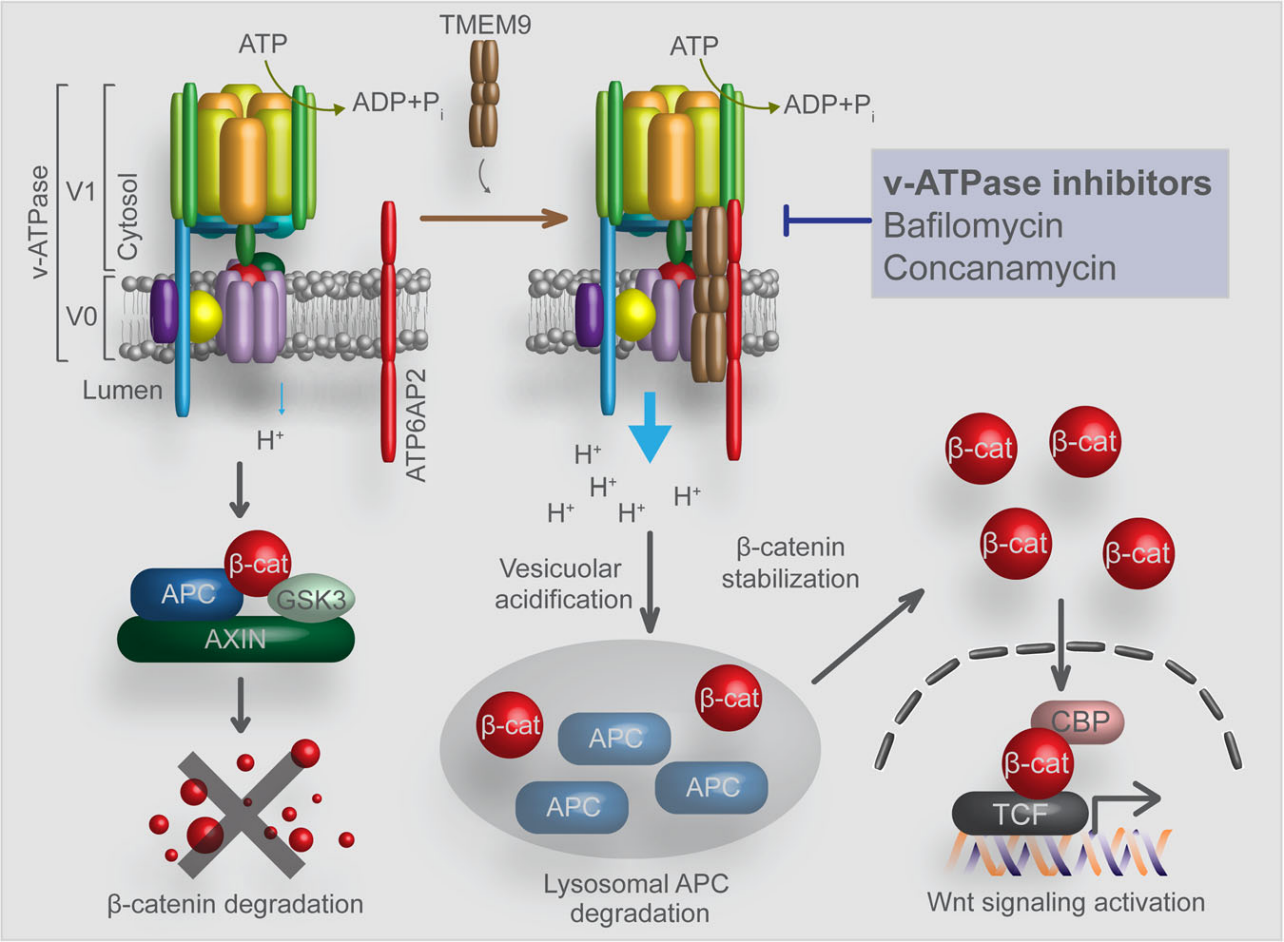
Inhibition of Wnt/β-catenin signaling activity by targeting the TMEM9-v-ATPase axis. TMEM9 expression is highly increased in CRC. As an amplifier of Wnt/β-catenin signaling, TMEM9 facilitates the assembly of v-ATPase, resulting in vesicular acidification and subsequent lysosomal degradation of APC. Then, the increased β-catenin transactivates Wnt target genes. The inhibition of TMEM9-v-ATPase-induced vesicular acidification by bafilomycin and concanamycin efficiently inhibits APC lysosomal degradation, which leads to the suppression of Wnt/β-catenin gene activation in cancer cells.

Conventional approaches targeting Wnt/β-catenin have led to various side effects, as mentioned above. Therefore, cancer-specific Wnt signaling regulators such as v-ATPase may be attractive molecular targets for Wnt signaling blockade. Chloroquine (CQ) and hydroxychloroquine (HCQ), inhibitors of lysosomes and autophagy, are clinically used for the treatment of diseases such as malaria and rheumatoid arthritis^145^. While the mechanism of action of CQ and HCQ is somewhat unclear, other v-ATPase inhibitors, such as bafilomycin (BAF) and concanamycin (CON), directly bind to and inhibit v-ATPase^146,147^. Compared with CQ and HCQ, BAF and CON showed marked inhibition of Wnt/β-catenin signaling in CRC. In addition, BAF and CON displayed an antiproliferative effect in CRC patient-driven xenograft and animal models without toxicity to normal cells and animals^39^. In addition, BAF and CON also strongly inhibit Wnt signaling activity in CRC cells, regardless of *APC* mutations. Thus, further research may lead to the development of not only safer but also more potent anti-v-ATPase drugs as cancer-specific Wnt/β-catenin inhibitors (Fig. 2).

## Conclusion

Genetic and epigenetic deregulation of Wnt/β-catenin signaling contributes to human cancer, which has led to the development of extensive approaches targeting Wnt/β-catenin signaling as cancer therapies. Nonetheless, the blockade of Wnt signaling impairs tissue homeostasis and regeneration, which needs to be resolved. Recent studies have identified several Wnt signaling regulators whose expression is specific to cancer cells. These cancer-specific regulatory processes of Wnt signaling may be druggable vulnerabilities of Wnt signaling-associated cancer.
